# Spinal Intradural Extramedullary Schwannomas Mimicking Intramedullary Tumors: A Case Series

**DOI:** 10.7759/cureus.110635

**Published:** 2026-06-10

**Authors:** Shichiro Katase, Rena Ogiu, Kazuhiro Tsuchiya, Miho Gomyo, Kazumasa Konishi, Naobumi Hosogane, Kenichi Yokoyama

**Affiliations:** 1 Department of Radiology, Kyorin University School of Medicine, Tokyo, JPN; 2 Department of Orthopaedics, Kyorin University School of Medicine, Tokyo, JPN

**Keywords:** cystic component, intradural-extramedullary tumor, intramedullary tumor, mri - magnetic resonance imaging, spinal schwannoma

## Abstract

Spinal schwannomas are among the most common intradural extramedullary tumors, together with meningiomas. Three cases of spinal schwannoma that presented predominantly with a cystic component and preoperative magnetic resonance imaging (MRI) findings mimicking those of intramedullary tumors were reported. The lesions were located at the C5-6, C7-T1, and T8-9 levels in one patient each. In all three patients, the lesions were located on the dorsal aspect of the spinal cord and compressed the spinal cord. MRI showed hyperintensity on T2-weighted images, with internal septal and nodular hypointense structures within the thoracic-level lesion. On T1-weighted images, hypointensity relative to the spinal cord was seen. In patients with lesions in the cervical and cervicothoracic level, postcontrast T1-weighted images revealed cystic components that showed no enhancement, while enhancement was observed along the cyst walls. In the patient with a lesion at the thoracic level, a cystic component and internal linear enhancement were seen, and the enhancement pattern differed from that observed in the other two cases. MR myelography (MRM) was performed in two patients, which helped delineate the extramedullary location of the lesions and their relationship with the spinal cord. In each case, a diagnosis of intradural extramedullary schwannoma was made based on surgical and pathological findings.

Many spinal schwannomas are intradural extramedullary and arise from a dorsal root of the spinal cord. Large lesions often show cystic degeneration and heterogeneous internal enhancement. In all three cases described, a cystic component was present, and enhancement consistent with a cyst wall was observed on postcontrast T1-weighted images. Internal linear enhancement was observed in the patient with a thoracic vertebral lesion. Although enlargement of the intervertebral foramen is frequently associated with spinal schwannoma, it was absent in these cases. Rather, these patients had intramedullary tumor-like findings. In conclusion, the presence of a cystic mass on the dorsal aspect of the spinal cord should raise suspicion for spinal schwannoma and may serve as a useful feature for differentiating it from an intramedullary tumor with cystic components. These three cases are valuable because they illustrate the characteristics of spinal schwannomas with cystic components.

## Introduction

Spinal tumors are commonly classified according to their relationship to the dura mater and spinal cord. Intramedullary tumors arise within the spinal cord itself, whereas intradural extramedullary tumors are located inside the dura but outside the spinal cord. This distinction is clinically important because it affects the differential diagnosis, surgical approach, and patient outcome.

Schwannomas are tumors that arise from Schwann cells. They occur in the peripheral nervous system and subcutaneous tissue, as well as in the central nervous system. Of the intradural extramedullary tumors, spinal schwannomas rank with meningiomas as the most frequently occurring benign tumors. Approximately 80% of spinal schwannomas show the morphology of intradural extramedullary tumors [1,2]. Spinal schwannomas most commonly occur in patients aged 40-60 years, with no sex predilection [3]. They are often located to extend through the intervertebral foramen, resulting in a dumbbell-shaped appearance. MRI findings of spinal schwannomas often consist of well-defined borders, hyperintensity on T2-weighted images, hypointensity on T1-weighted images, and clear enhancement on postcontrast T1-weighted images. However, larger tumors frequently show cystic degeneration and may demonstrate heterogeneous internal signal intensity on MRI [1]. Moreover, if the tumor has extensive contact with the spinal cord, its border with the spinal cord may be poorly defined, and it may be necessary to differentiate it from an intramedullary tumor. In the three cases reported, the spinal schwannomas showed extensive spinal cord contact and cystic degeneration internally. We present their findings and discuss diagnostic points that differentiate them from intramedullary tumors on MRI.

## Case presentation

Case 1

A woman in her 20s was referred from another hospital with neck pain and left arm numbness. She had no significant past medical history or family history. Examination on presentation identified no problem with cervical range of motion; left arm numbness was in the region innervated by the fifth and sixth cervical nerves. No symptomatic improvement was seen with physical therapy, so an MRI of the cervical spine was performed. On T2-weighted sagittal images, a mass lesion measuring approximately 2.4 × 0.9 cm was seen on the dorsal aspect of the spinal cord at the level of the fifth and sixth cervical spine. The lesion showed internal hyperintensity and a cystic component (Figure 1A). On T1-weighted images, the lesion showed internal hypointensity relative to the spinal cord (Figure 1B).

**Figure 1:**
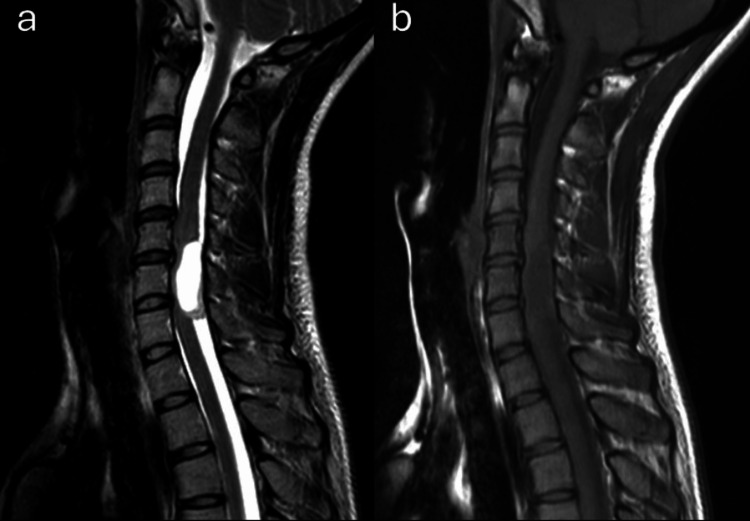
Case 1: T2-weighted sagittal image (A) and T1-weighted sagittal image (B) A cystic mass measuring approximately 2.4 × 0.9 cm and showing internal hyperintensity is seen on the dorsal aspect of the spinal cord at the level of C5-6 (A). The inside of the mass shows hypointensity relative to the spinal cord (B).

On T2-weighted axial images, the mass was seen to be located on the left dorsal aspect of the spinal cord, strongly compressing the spinal cord. Its border with the spinal cord was ill-defined (Figure 2A). MR myelography (MRM) showed the mass to be hyperintense internally, with no evident enlargement or deformation of the nerve root sleeve contiguous with the mass (Figure 2B). PostcontrastT1-weighted images showed little enhancement in the central portion of the mass, with enhancement observed predominantly along the cyst wall (Figures 3A-3B).

**Figure 2:**
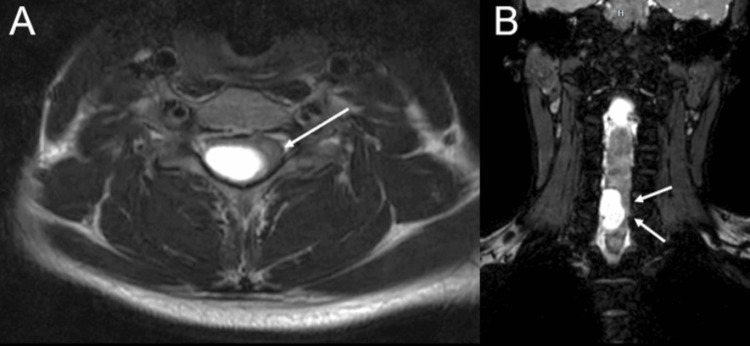
Case 1: T2-weighted axial image (A) and 3D MRM source image (B) A cystic mass is seen on the right dorsal aspect at the level of C5-6 (arrow), and the intervertebral foramina are not enlarged (A). The normal spinal cord is seen displaced to the left side (arrows) (B). MRM: magnetic resonance myelography

**Figure 3:**
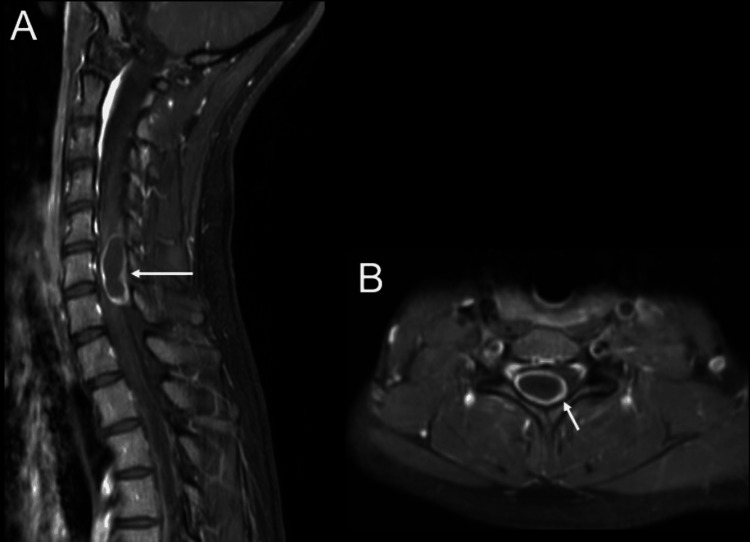
Case 1: Postcontrast T1-weighted sagittal (A) and axial (B) magnetic resonance images Enhancement of the cyst wall is seen (arrows).

Based on the imaging findings, a laminectomy and tumor resection were performed with a preoperative differential diagnosis of an intramedullary or an intradural extramedullary tumor. Intraoperatively, the tumor was identified as an intradural extramedullary tumor. Moreover, the tumor was diagnosed to have arisen from the ventral root of the right sixth cervical nerve. Gross examination revealed an encapsulated, yellowish-white tumor measuring 2.4 × 1.6 × 0.5 cm, with internal cystic degeneration and hemorrhage. Histologically, mildly atypical spindle-to-stellate tumor cells proliferated in fascicles with alternating loose and dense cellular areas. Numerous small blood vessels and stromal inflammatory cell infiltration, predominantly lymphocytic, were observed. The final pathological diagnosis was schwannoma. The patient’s symptoms improved postoperatively, and no recurrence has been observed.

Case 2

A man in his 40s with a six-month history of left shoulder pain, left precordial chest pain, and stiffness in his left hand presented to our hospital because the symptoms had not improved. Physical examination showed muscle weakness in the left upper limb. In addition, the patient felt pain in both shoulders during neck anteflexion. T2-weighted sagittal MRI of the cervical spine showed a hyperintense mass lesion measuring approximately 2 × 0.8 cm on the dorsal aspect of the spinal cord at the level of the first thoracic spine (Figure 4A). T1-weighted images showed internal hypointensity (Figure 4B).

**Figure 4:**
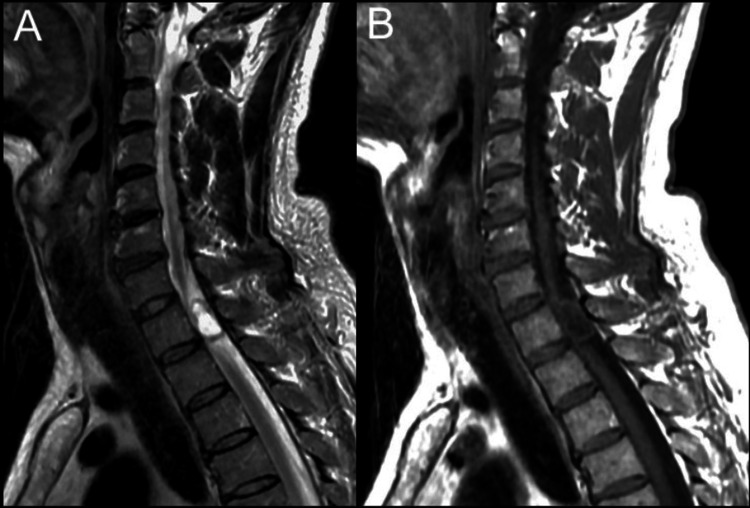
Case 2: T2-weighted sagittal image (A) and T1-weighted sagittal image (B) A cystic mass measuring approximately 2 × 0.8 cm is seen on the dorsal aspect of the spinal cord at the level of T1. The mass shows internal hyperintensity (A). The central part of the tumor shows hypointensity relative to the spinal cord (B).

T2-weighted axial images showed the tumor extending from the dorsal midline to the left side of the spinal cord and compressing it. In addition, a hypointense area was seen at the tumor border (Figure 5A). Postcontrast T1-weighted sagittal images showed a mass with a cystic component, no internal enhancement, and predominant enhancement along the cystic wall (Figure 5B). Tumor resection was subsequently performed. Intraoperatively, the tumor was found to be an extramedullary mass and proved to be a schwannoma arising from the dorsal root of the left 1st thoracic nerve. Histologically, spindle-shaped cells proliferated in a loose-to-dense arrangement, with nuclear palisading and Verocay bodies.

**Figure 5:**
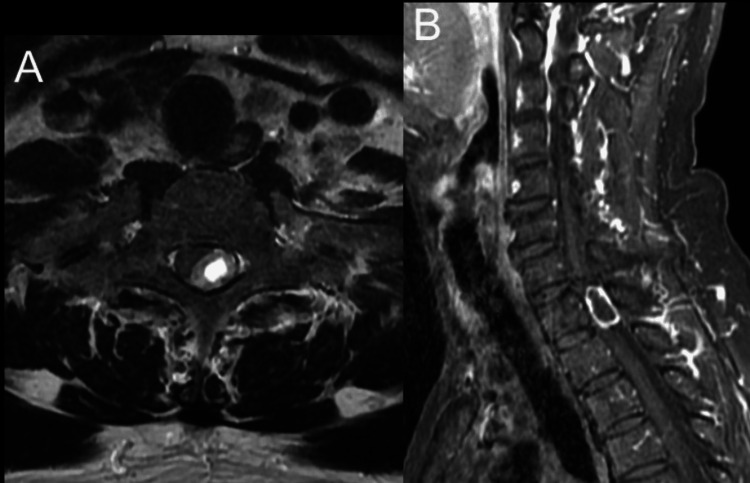
Case 2: T2-weighted axial image (A) and postcontrast T1-weighted sagittal image (B) The tumor is seen on the left dorsal aspect of the spinal cord. The tumor wall appears thick and hypointense (A). Enhancement consistent with a cyst wall is seen (B).

Case 3

The patient was a man in his 50s who presented with gait disturbance and subsequent numbness in both lower limbs. The exact time of symptom onset was unclear. On presentation, the patient was walking with a cane. Neurological examination showed numbness and hypesthesia of the toes. No bladder or bowel dysfunction was observed. After a short period of physical therapy, no symptomatic improvement was noted; an MRI of the thoracic spine was performed. T2-weighted sagittal images showed a hyperintense mass lesion measuring approximately 1.4 × 1.1 cm on the left dorsal aspect of the spinal cord at the level of the eighth and ninth thoracic spine (Figure 6A). On T1-weighted sagittal images, the inside of the tumor was hypointense compared with the spinal cord (Figure 6B).

**Figure 6:**
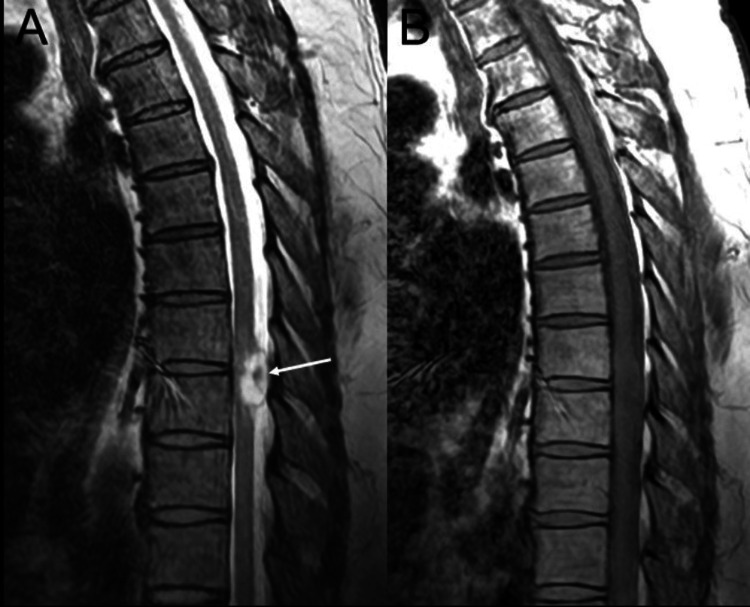
Case 3: T2-weighted sagittal image (A) and T1-weighted sagittal image (B) A party cystic mass measuring approximately 1.4 × 1.1 cm and showing internal hyperintensity is seen on the dorsal aspect of the spinal cord at the level of T8-9. A nodular and septate hypointense area is seen in the central region (arrow) (A). The inside of the mass shows mild hypointensity relative to the spinal cord (B).

On T2-weighted axial images, the tumor showed hyperintensity internally, where a hypointense septal and nodular structure was seen. The border between the spinal cord and the tumor was poorly defined; the normal spinal cord showed a rightward deviation (Figure 7A). On MRM, the tumor compressed the normal spinal cord, with cord-like and nodular structures visualized as hypointense areas within the tumor (Figure 7B). Postcontrast T1-weighted images showed enhancement mainly at the tumor margin. Moreover, contrast enhancement was observed even in septal and nodular structures that showed hypointensity on T2-weighted images (Figures 8A-8B). The tumor was subsequently resected. Based on the intraoperative findings, the mass was found to be an intradural extramedullary tumor arising from the dorsal root of the left eighth thoracic nerve. Pathological diagnosis showed it to be a schwannoma.

**Figure 7:**
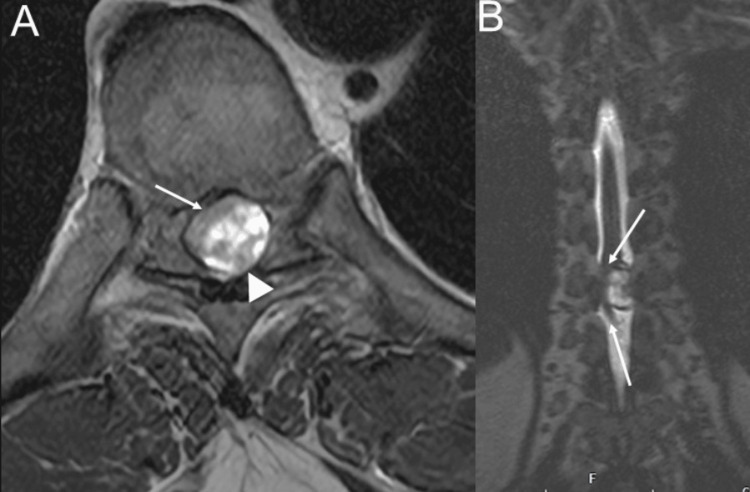
Case 3: T2-weighted axial image (A) and 3D MRM (source image) (B) The border with the normal spinal cord is ill-defined; the normal spinal cord is seen on the right ventral side (white arrow). The tumor is seen on the left dorsal aspect of the spinal cord, with a nodular and septate hypointense area seen internally (arrowhead) (A). The tumor is on the left side of the spinal canal, and the normal spinal cord is displaced to the right (arrow) (B). MRM: magnetic resonance myelography

**Figure 8:**
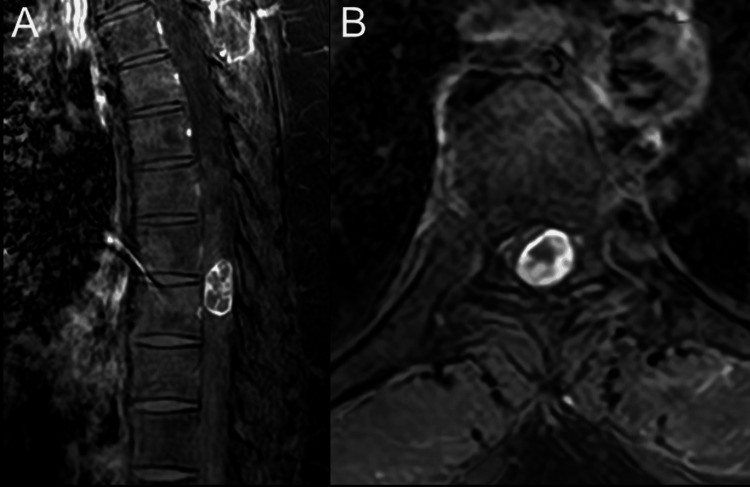
Case 3: postcontrast T1-weighted sagittal (A) and axial (B) magnetic resonance images Enhancement of the cyst wall is seen, and nodular and septate enhancement is seen internally (A, B).

## Discussion

Schwannomas are benign tumors that arise from Schwann cells. Nearly all spinal schwannomas are intradural extramedullary tumors, with intramedullary schwannomas reported to account for approximately 1% of cases [1]. Spinal schwannomas most commonly occur in the cervical and thoracic spine but can arise at any spinal level [4]. They are thought to arise frequently from dorsal roots of the spinal cord, which are afferent sensory nerves. Consequently, tumor enlargement often gives rise to radicular pain, resulting in symptoms such as lower limb numbness and radiating thoracodorsal pain [5,6]. With enlargement, the tumor frequently extends through the intervertebral foramen into the paraspinal space, resulting in a characteristic dumbbell- or hourglass-shaped configuration.

MRI findings of spinal schwannoma often include a well-circumscribed tumor, hyperintensity on T2-weighted images, iso- or hypointensity on T1-weighted images, and enhancement on postcontrast T1-weighted images. On T2-weighted images, the central region may be hypointense, a feature referred to as the target sign, which is a frequent finding in schwannomas [7]. Larger lesions often show cystic degeneration on MRI, with heterogeneous signal intensity and enhancement seen predominantly at the tumor border on MRI.

Schwannomas are classified histologically into Antoni A and Antoni B types [1,8]. Antoni A type is hypercellular and shows nuclear palisading, with spindle-shaped nuclei arranged in parallel rows, forming Verocay bodies. Antoni B type is hypocellular and has a loose stroma with degenerative or edematous changes. Cystic degeneration may be related to coalescence of small cystic components, particularly in Antoni B types, as well as reduced blood flow, ischemia, hemorrhage, or necrosis due to degenerative vascular changes [5,8,9]. In our three cases, the pathological findings showed a mixture of Antoni A and Antoni B types, with no obvious differences among the cases. Although the tumor arose from a ventral root in one case and from dorsal roots in the other two cases, continuity with the spinal nerve root could not be identified on preoperative imaging.

Intramedullary tumors were suspected in the present cases based on the initial MRI findings. Intramedullary tumors with cystic components include spinal astrocytomas and ependymomas. Spinal astrocytomas are common intramedullary tumors in childhood but are also frequently seen in adults. They typically show hyperintensity on T2-weighted images, often extend in the craniocaudal direction, and may be eccentrically located within the spinal cord. Spinal pilocytic astrocytomas in particular may contain cystic components within or around the tumor [10,11]. Ependymomas arise from ependymal cells of the central canal and therefore tend to have a central location within the spinal cord; they may also contain cystic components. Myxopapillary ependymomas usually occur in the cauda equina or filum terminale and may present as extramedullary tumors [11].

In the present three cases, although the tumors were extramedullary schwannomas with cystic components, the borders with the spinal cord were poorly defined, and typical imaging features of spinal schwannoma, such as enlargement of the intervertebral foramen, were not observed. Therefore, cystic extramedullary schwannomas with a poorly defined border with the spinal cord may show imaging findings resembling those of intramedullary tumors. Such lesions should be considered in the differential diagnosis, particularly when located on the dorsal aspect of the spinal cord, because spinal schwannomas often arise from dorsal nerve roots as mentioned above. Spinal cord edema associated with tumor compression may also mimic an intramedullary tumor; therefore, careful evaluation is required in such cases [12].

From a practical diagnostic perspective, the present cases suggest several imaging clues that may help identify similar lesions. These include a cystic mass located on the dorsal aspect of the spinal cord, poor delineation of the tumor-cord interface, displacement of the normal spinal cord rather than intrinsic spinal cord swelling, absence of intervertebral foraminal enlargement, and enhancement predominantly along the cyst wall. Although none of these findings are specific on its own, their combination should prompt consideration of an intradural extramedullary schwannoma, even when the lesion initially appears to be intramedullary.

Thin-slice T2-weighted images and MRM centered on the tumor are considered helpful for differentiating these lesions from intramedullary tumors. MRM source images acquired in 3D may be particularly of diagnostic value. The images should be carefully evaluated for the interface between the tumor and normal spinal cord, continuity with a nerve root, and enlargement of the subarachnoid space above and below the tumor. However, in some cases, distinguishing continuity between the tumor and the nerve root may be difficult due to limitations in spatial resolution of MRI. As noted above, meningiomas, like schwannomas, are common intradural extramedullary tumors; however, meningiomas rarely undergo cystic transformation, and the dural tail sign on postcontrast T1-weighted images may help differentiate them from schwannomas [13].

The present three cases demonstrate that cystic extramedullary schwannomas can mimic intramedullary tumors, particularly when located on the dorsal aspect of the spinal cord and with a poorly defined border with the spinal cord. Recognition of this imaging pattern may help include extramedullary schwannoma in the differential diagnosis and guide appropriate surgical planning.

## Conclusions

We described three cases of extramedullary schwannomas with cystic components that mimicked intramedullary tumors. We consider that distinguishing between intramedullary and extramedullary tumors based on diagnostic imaging of spinal cord tumors is particularly important for determining a treatment plan and surgical approach. Moreover, for tumors located on the dorsal aspect of the spinal cord, an extramedullary schwannoma should be considered in the differential diagnosis, even when an intramedullary tumor is suspected. An associated cystic component may be a useful imaging clue for considering spinal schwannoma in the differential diagnosis.
